# The Costs and Benefits of Employing an Adult with Autism Spectrum Disorder: A Systematic Review

**DOI:** 10.1371/journal.pone.0139896

**Published:** 2015-10-07

**Authors:** Andrew Jacob, Melissa Scott, Marita Falkmer, Torbjörn Falkmer

**Affiliations:** 1 School of Occupational Therapy & Social Work, Curtin University, Perth, Western Australia, Australia; 2 Cooperative Research Centre for Living with Autism (Autism CRC).Long Pocket, Brisbane, Queensland, Australia; 3 School of Education and Communication, CHILD programme, Institute of Disability Research, Jönköping University, Jönköping, Sweden; 4 Rehabilitation Medicine, Department of Medicine and Health Science (IMH), Faculty of Health Sciences, Linköping University, Linköping, Sweden; Harvard Medical School, UNITED STATES

## Abstract

**Background:**

Despite an ambition from adults with Autism Spectrum Disorder (ASD) to be employed, there are limited opportunities for competitive employment for this group. Employment is not only an entitlement enjoyed by others in society, but employing adults with ASD also has economic benefits by decreasing lost productivity and resource costs for this group. Few studies have explored the cost-benefit ratio for employing adults with ASD and even fewer have taken the viewpoint of the employer, particularly applying this situation to ASD. Until such study occurs, employers may continue to be reluctant to employ adults from this group.

**Objective:**

This review aimed to examine the costs, benefits and the cost-benefit ratio of employing adults with ASD, from a societal perspective and from the perspective of employers.

**Methods:**

Eight databases were searched for scientific studies within defined inclusion criteria. These databases included CINAHL Plus, Cochrane Library, Emerald, Ovid Medline, ProQuest, PsycINFO, Scopus and Web of Science.

**Results and Conclusion:**

Enhancing the opportunities for adults with ASD to join the workforce is beneficial from a societal perspective, not only from an inclusiveness viewpoint, but also from a strict economic standpoint. Providing supported employment services for adults with ASD does not only cut the cost compared with providing standard care, it also results in better outcomes for adults with ASD. Despite the fact that ASD was the most expensive group to provide vocational rehabilitation services for, adults with ASD have a strong chance of becoming employed once appropriate measures are in place. Hence, rehabilitation services could be considered as a worthwhile investment. The current systematic review uncovered the fact that very few studies have examined the benefits, the costs and the cost-benefit ratio of employing an adult with ASD from the perspective of employers indicating a need for this topic to be further explored.

## Introduction

Autism Spectrum Disorder (ASD) is a lifelong condition that represents individuals who experience similar characteristics that affect their behaviour patterns [1]. The term ASD traditionally includes the diagnoses of Autistic disorder, Asperger syndrome (AS), Childhood Disintegrative Disorder, Rett Syndrome and Pervasive Developmental Disorder Not Otherwise Specified (PDD NOS) [2]. In the two major diagnostic manuals,: DSM- IV and ICD–10 [2, 3] ASD is defined by impairments in three domains; social reciprocity, communication and behaviour and interests [2].Diagnosis requires reduced functionality across all, or at least two of the three domains. ASD comprises heterogenic phenotypes and hence, the severity of symptoms within each domain can differ greatly between individuals [4]. For example, individuals diagnosed as having AS are expected to have developed typically in regard to early language development and cognitive abilities [2] and the term high functioning Autism Spectrum Disorder (HFA) is commonly used when referring to individuals with ASD with a mean IQ score within or above the normative average range [5]. For the purpose of this article, the term ASD will be used throughout to represent adults with HFA/AS, who do not have an intellectual disability.

Despite the fact that most adults would have been diagnosed according to DSM-IV, it is worth noting that in the new diagnostic manual DSM–5, the separate diagnoses mentioned above are merged into one single category, ASD [6]. The diagnostic criteria in the DSM–5 focus on the severity of limitations in the core domains, i.e., social communication, social interaction, restricted interests and repetitive behaviours, and do not define specific diagnostic groups. Furthermore, DSM–5 recognises that sensory differences are common in ASD and hence, unusual sensory behaviours are now included in the domain of stereotyped motor and verbal behaviour [6].

Difficulties in Theory of mind, which comprise the ability to assess one self’s and other’s intentions, actions and behaviours, have been used in order to explain some manifestations in ASD [7–10]. Social communication for example, requires the ability to identify intention in others, ven individuals diagnosed with AS may have difficulties that affect their social communication style and ability to interpret non-verbal and spoken language [11]. It is common that individuals with ASD experience difficulties in the area of executive functions, i.e., in cognitive processes, such as working memory, planning, initiating and monitoring actions, impulse control, and mental flexibility [8, 12, 13]. Furthermore, individuals with ASD have been purported to have a perceptual processing style biased towards attending to detail as a result of a weak central coherence[14, 15]. A detailed processing of stimuli may explain the perceptual sensitivity [16, 17] and together with difficulties in executive functioning, may result in commonly experienced difficulties in generalisation, high reliance on aroutines and sameness [16].

The number of individuals being diagnosed with ASD in the United States (US) has been increasing annually at 10–17% [18]. In Australia, the prevalence of Autism is estimated at 24.2 to 47.2 for every 10,000 people and the prevalence of AS at 12.7 to 15.3 for every 10,000 people with an overall figure of 36.9 to 62.5 per 10,000 [19]. The increase in the prevalence of ASD has, to a large part, been explained by a change in diagnostic criteria, a greater awareness of the characteristics associated with ASD in clinicians and improved possibilities to diagnose ASD in young children [20, 21]. However, it has also been suggested that the fact that eligibility for services and support is often based on a categorical diagnosis in western countries may have contributed to the increased prevalence, since it may result in a tendency to diagnose children despite insufficient symptoms in order to fulfil all required criteria of ASD [22]. Nonetheless, with the existing prevalence statistics, there are approximately 153,000 adults with ASD in Australia of working age (16–64 years) [23].

Becoming and remaining employed in a competitive job market can be challenging for any individual, yet can be especially complex for adults with ASD because of the social interaction and communication needs in the workplace [18]. However, to date, most research in ASD focuses on early intervention for children with ASD, while only a limited amount exists for adults and adolescents with ASD as they transition into the workforce [18]. Competitive employment (henceforth employment and employed in the current study refer to competitive employment if not otherwise specified), is one of the key ambitions for most people after their education is completed, this is also true for adults with ASD [18]. Despite a need and ambition of individuals with ASD to become employed, there are limited opportunities for competitive employment [18]. This may be attributed to interactional difficulties experienced by adults with ASD, impacting on their ability to find and maintain employment. Difficulties in social interaction and communication in the workplace impact on job performance such as: effectively interacting with supervisors and co-workers, understanding and interpreting social rules, difficulty working independently and resistance to workplace change [24, 25]. As result of these interactional difficulties, the jobs of adults with ASD are often terminated prematurely [26]. The consequences of unemployment remain an important issue as work provides an opportunity to improve quality of life in adults with ASD and encourages personal dignity, as well as increased cognitive performance [18]. Alternatively, unemployment often contributes negatively to an individual’s quality of life by increasing social isolation and creating a lack of cognitive and mental stimulation [18].

The characteristics of ASD, in particular social and communication difficulties, may present challenges to individuals in the workplace that may require managers and co-workers to develop previously unused skills. These skills may include; overcoming communication difficulties between themselves and employees/colleagues with ASD, considering the need for possible supervision, and providing clarity to these individuals around social rules in the workplace. Employers and co-workers also need to consider possible subdued or exaggerated responses to sensory stimulation from adults with ASD, as well as allowing time for adjustment for change within the workplace [27].

Nevertheless, as mentioned, the desire and ability to become employed and build a meaningful life exists for adults with ASD. Therefore, thoughtful consideration needs to be given to their unique capabilities and characteristics. This includes; considering each adult’s strengths, challenges and personal interests, which ultimately can lead to appropriate job-matching for employment. Effective job-matching between the person and their environment, when combined with the use of proper supports [28], allows an adult with ASD to be productive and valuable to their employers, ultimately maintaining their ongoing employment [18]. Additionally, the management practices of the employer, including a willingness to supply necessary accommodations and flexibility, have been shown to be important for the success of adults with ASD, in the workplace [28]. Employers who foster an atmosphere of mutual support and a worker-friendly environment that benefits both the worker with ASD and the employer create a more inclusive workplace and ultimately a more inclusive community [28].

The number of working age adults with ASD in Australia is expected to increase over the next 10 years to 181,000 [23]. Due to this rapidly increasing number of individuals with ASD now graduating from high school, there is a growing need and increasing attention from educational researchers to understand what supports and what hinders employment outcomes for students with disabilities [18]. Not only is access to employment an entitlement enjoyed by other groups in society that enhances their quality of life and dignity, but employment also allows an adult to receive wages that can be put towards supporting themselves and building their own future [18]. Employment can also create a sense of purpose, meaning, independence and identity for an individual, from which adults with ASD could benefit and thrive. In addition employing adults with ASD has economic benefits for both employers and governments.

Even for adults who have received a postsecondary education, becoming employed remains problematic [18] as it is believed that 50–75% of adults with ASD are unemployed [29–31]. This is unfortunate, as the wages from employment allow adults with ASD to be financially self-reliant, decreasing reliance on government payments [32]. Hence, employing individuals with ASD may decrease the cost of community supports, such as adult care and day time activities. Additionally, overlooking the potentially valuable contribution of employees with ASD results in lost productivity, which has been approximated to cost Australia between AUD$ 939–1,357 million per annum [19]. Hence, the societal financial impact of adults with ASD without employment may actually harm the economy [10]. However, the advantages employers receive from employing adults with ASD are yet to be examined and identified [18]. Benefits can include; reliability, lower levels of absenteeism, trustworthiness, attention to detail, a high degree of accuracy in visual tasks, advantageous long-term memory and concentration ability [18]. This is in addition to productivity benefits, including the greater work ethic and better focus that individuals with ASD apply to roles and jobs that might be repetitive in nature or are isolated from others and which other workers may be reluctant to perform [18].

The amount of research that considers employment of individuals with ASD from the perspective of employers is minimal. The research is particularly limited in understanding the question “is hiring an adult with ASD financially cost effective for an employer?” Previously, studies have explored the financial costs and benefits of supported or competitive employment from the viewpoint of the worker, taxpayer, government and society, but no study exists that explores the cost-benefit ratio from the viewpoint of the employer in relation to employees with ASD [33]. This gap in the research may contribute to employers’ concerns about having to pay for extensive work training, continual supervision and other expensive accommodations when they employ an adult with ASD [33]. Additionally, there is no current evidence available that identifies if there is a worthwhile financial cost-benefit ratio to the employer’s business from hiring an adult with ASD in terms of productivity. Until these relevant and valid concerns from employers are addressed, there is a potential for employers to show reluctance in employing adults with ASD [33]. Hence, the aim for the current systematic review was to examine the costs, benefits and the cost-benefit ratio of employing adults with ASD, from a societal perspective and from the perspective of employers.

## Methods

Eight databases were filtered for scientific studies within the set inclusion criteria. These databases included CINAHL Plus, Cochrane library, Emerald, Ovid Medline, ProQuest, PsycINFO, Scopus and Web of Science. The key terms were refined with truncation expansion with assistance from librarian staff and included: autis*, ASD, ASC, aspergers*, “high functioning”, employ*, hiring*, job*, occupt*, activity*, cost*, benefit*, economic*, cost effectiveness, “cost benefit”, analysis. When using Boolean operators the following combined search strategy used in review was autis* OR ASD OR ASC OR aspergers* OR “high functioning” AND employ* OR hiring* OR job* occupt* OR activity* AND cost* OR benefit* OR economic*. A screening for relevant titles and abstracts was completed. After this, a full text review of remaining articles was initiated and a manual search was completed to select articles from reference lists of collected articles.

### Inclusion/Exclusion Criteria

Articles that specifically mentioned ASD were included in the results. This review accepted articles that focused on the different types of ASD including: ASD, AS and HFA These articles were kept if they were from 1994 onwards due to the creation of the DSM-IV [1]. Further inclusion criteria included articles that described adults aged 18 years and over. A linguistic limit was applied for articles to be included only if published in English. The hierarchy of evidence was used as a guide in determining the level of evidence for articles [34]. This systematic review included all forms of peer reviewed articles. Textbooks and similar grey literature were excluded.

Studies were included if they focused on competitive employment for adults with ASD including casual employment, i.e., the employee is only being paid for the time actually worked and does not receive payment for public holidays personal/carer’s leave or annual leave, part time competitive employment, full time competitive employment and supported employment. Supported employment in this review includes employment obtained through programs that support a person with disability with the process of finding and retaining a job in the open job market.

Outcomes were grouped according to costs of employing adults with ASD, benefits of employing adults with ASD and cost-benefits of employing an adult with ASD. Exclusion criteria included: children with ASD and participants in studies with significant comorbidities. After consideration of 10 randomly selected journal abstracts by two reviewers using the preselected inclusion and exclusion criteria, 100% agreement was found. Issues concerning eligibility for the articles were resolved with discussion and a mutual agreement settled upon.

### Methodological Quality

The full text articles were assessed for quality using the Kmet checklist (S2 Table) [35]. The Kmet checklist has a 14 point list with scoring criteria. Scores were represented as percentages with the strength of the evidence being categorised as, strong (> 80%), good (70–80%), adequate (50–70%) or limited (<50%). The assessment of the included studies using the Kmet Form was completed independently by two of the authors who reached a consensus on all articles. The scores and description of methodological quality are displayed in S1 Table.

### Data Extraction

The Cochrane Handbook for Systematic Reviews Section 7.3 was used as a guide to create a data extraction form (S3 Table) [36]. The data extraction form included citation, publication status, database, level of evidence, study design, population, methods, and outcomes grouped according to costs of employing adults with ASD, benefits of employing adults with ASD and cost-benefits of employing an adult with ASD and results and conclusions from the studies.

### Data Synthesis and Analysis

The analysis and synthesis of the themes included: the cost effectiveness of employing an adult with ASD to governments, the cost effectiveness of employing adults with ASD to society and the cost effectiveness of employing these adults with ASD to employers. To discuss the themes from the review a narrative approach was applied.

## Results

**Fig 1:**
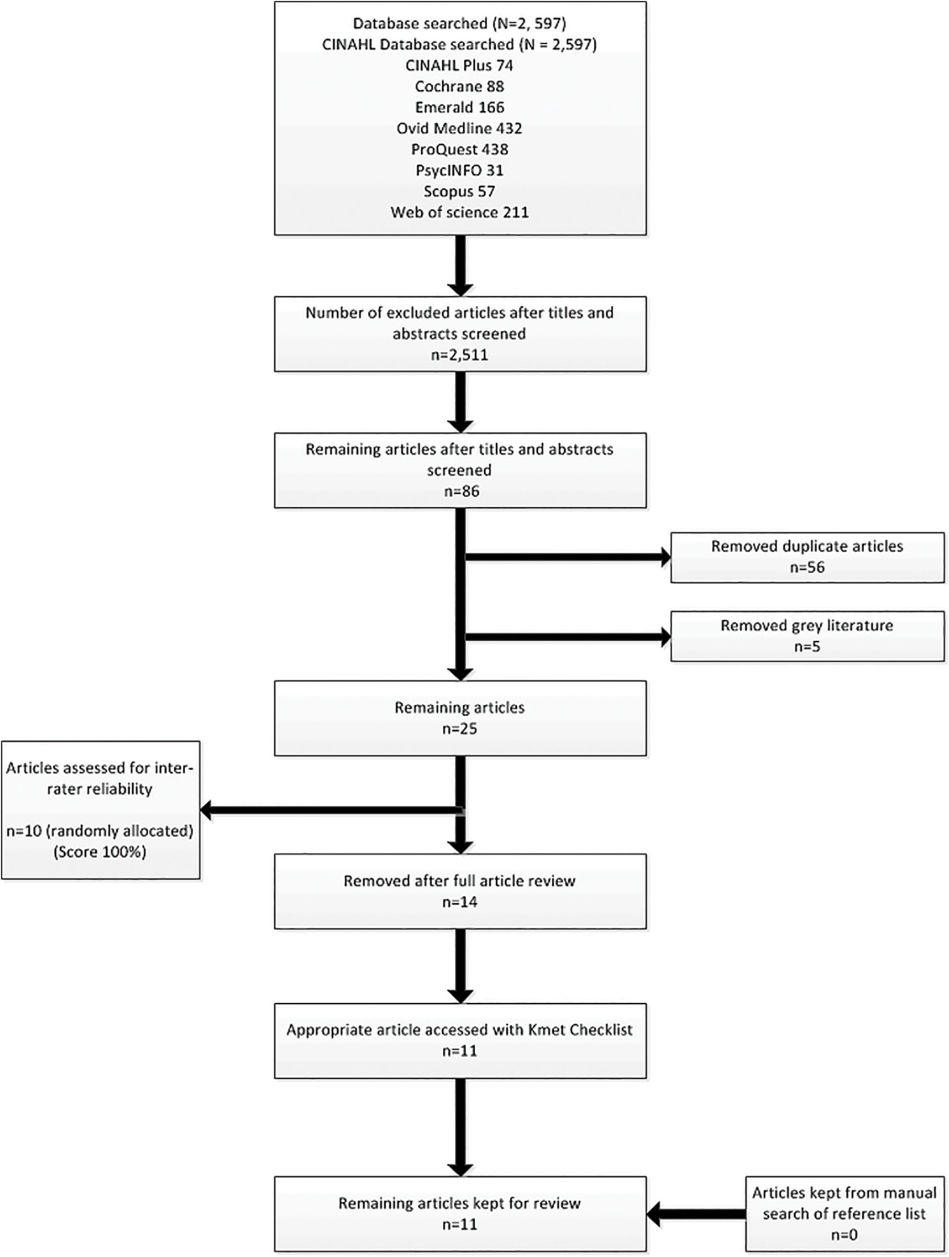
Flow Diagram for Selection of Studies

After searching electronic databases, 2,597 titles were found. After filtering ofthe titles and abstracts, 2,511 were excluded leaving 86 articles. Duplicates articles (56) were removed as well as five grey literature articles. The remaining 25 articles were then retrieved in full. Fourteen articles were excluded after full paper review as they did not fulfil the inclusion criteria, leaving 11 articles. The reference lists of these articles were then manually examined for suitable studies, with none being identified. The Kmet Form was then used to rate the remaining 11 articles to determine their methodological quality. This process is displayed in Fig 1.

The 11 articles that met the inclusion criteria had a total number of 67,251 participants included. There were also two studies that used information from national databases rather than using participants [37, 38]. The study designs of the included articles were four cohort studies [37, 39–41], three case control studies [42–44], three descriptive studies [32, 38, 45] and one correlational study [46]. Of these included studies, four described the costs to governments for employing these adults with ASD [32, 41, 43, 44] and four explored the costs to society when employing adults with ASD [37, 38, 40, 45]. The remaining three studies explored the cost-benefits to the employer of adult/s with ASD [39, 42, 46].

### Quality Assessment of Studies

The methodological quality of studies assessed with the Kmet Form, ranged from adequate to strong, as described in S2 Table. Eight of the included studies were identified as strong [38–43, 45, 46]. The remaining three were rated as adequate according to the Kmet [32, 37, 44].

Jӓrbrink, McCrone (32) used interviews with subjects’ relatives to obtain information regarding costs. The study was limited to four communities in Sweden and made no mention of the confounding factors in the study. The study used a limited sample of 19 participants limiting transferability. In the study of Mavranezouli, Megnin-Viggars (44) limitations existed in the area of random allocation of the participants and in this study there was no mention of controlling for confounding factors. In the study by Ganz (37) the methodology was not well suited as it used a prevalence-base cohort or hypothetical cohort to describe the costs of employing an adult with ASD, which limits the ‘ general application of the findings.

### Intervention

The intervention was employment for adults with ASD. Casual, part time, full time competitive employment and supported employment were all included. Employment was considered using a cost-benefit analysis, in order to predict the cost and benefits of employing adults with ASD for the employer. It also predicted the cash flow during a set period. These costs include dollars spent, as well as indirect dollars allocated on other areas to achieve change.

### Findings

#### Costs to governments

A total of four studies explored the cost of ASD to the government. The study by Howlin, Alcock (41) explored a group of participants diagnosed with ASD who had been involved in a supported employment program. This study assessed the outcome from supported employment over an eight-year period. The results from this study showed that the overall savings to the Exchequer over an eight-year period were in total £179, 095 for the 114 jobs in the program, which can be seen in relation to a reduction in benefits and national insurance and increased tax contribution. A significant decrease in the number of benefits received was also observed once these participants were employed (median pre-work = £2907, range £0–£9193; median post-work = £0, range £0–£6801; median reduction = –£1974, range +£1440 to –£9030; Wilcoxon *Z* = –7.72; *p* < 0.001). The results of this study demonstrate that employing individuals with ASD can save government costs, through reducing the number of benefits people with ASD require when unemployed.

Between 2000 and 2003 a study was conducted in four communities in western Sweden [32]. The study aimed to improve authorities’ understanding of people with ASD and their need for employment. Results from this study found the average annual community support cost for each young adult with ASD was €7154. It was also found that employment support accounted for 4.0% of the total annual cost, while community support represented 22.6% of total annual cost (€596 per month per participant). The daily activities costs accounted for 20.9% of the baseline total service cost or €310 per person per month. The employment services cost represented 2.6% of the total cost for these participants, while the average annual informal care cost was approximated at €1554 and expenses at €1052. Informal care costs represented 8.2% of total costs. These results show that if more individuals were employed, it would reduce the amount spent on daily activities and carers for the government.

Cimera, Wehman (43) conducted a study of people with ASD who attended sheltered workshops before entering supported employment programs, to determine if they had better outcomes than those who did not receive sheltered employment services. This study found no differences between these groups for employment rates. Adults previously in sheltered workshops received lower wages (US$129.36 compared to US$191.42 per week), and were more expensive to serve (US$6,065.08 compared to US$2,440.60), compared with the group who had not been in sheltered workshops prior to supported employment. This study concluded that individuals with ASD had better vocational outcomes if they did not enrol in sheltered workshops before entering supported employment. This showed that vocational rehabilitation costs for individuals with ASD in sheltered employment prior to participating in supported employment were greater when compared to adults with ASD who only participated in supported employment.

The cost-effectiveness in the United Kingdom (UK) of supported employment versus day services for adults with ASD was again supported by Mavranezouli, Megnin-Viggars (44). They demonstrated that adults with ASD in supported employment had better outcomes in comparison to standard care for adults with ASD. Providing supported employment only added an extra cost of £5,600 per quality-adjusted life year or £18 per additional week in employment. These findings agree with those of Cimera et al. [43] supporting that there can be a financial gain for the government to provide supported employment services.

#### Cost to society

A total of four studies explored the cost of ASD to society [15,16,18,23]. An estimation of the economic significance of ASD in the UK, found that the mean annual costs (including lost employment, but excluding benefits), for an adult with ASD and an additional intellectual impairment living in family households was £36,507, in supported accommodation £87, 662, in residential care £88,937 and in long term hospital care £97,863 [38]. This research also determined that for adults with ASD without an intellectual disability living in a family household, the annual cost was £ 32,681, a major element being the cost of lost productivity for society and tax revenue for the Exchequer and lost employment for the adult with ASD. The aggregate national UK cost was £25 billion. The cost of supporting adults with an intellectual impairment (including lost employment) represents two-thirds of these costs (£17 billion). Publicly funded services accounted for 59% of this total, with lost employment for the adult with ASD (36%) and family expenses (5%) accounting for the remainder. This research also reported that amongst individuals with ASD without an intellectual disability, this group has an annual cost of £32,681 of which the greatest part was attributed to lost employment and lost productivity, when they could alternatively be employed and contributing a valuable return to society and tax revenue.

The employment outcomes and service costs for adults with ASD was examined by a study of the US vocational rehabilitation system during the period of 2002–2006 [40]. It found that during 2002, the cost of providing vocational rehabilitation services to an individual with ASD was US$3,282 per person. In 2006 this decreased to US$2,992 per person. This was in comparison to the costs of providing the same services to the overall vocational rehabilitation population, which conversely increased from US$2,263 to US$2,336 during the same time period. The research also noted that in relation to wages earned by people with ASD, during 2002 this group cost the vocational rehabilitation US$26.74 for every dollar these individuals earned in wages. In 2006 this ratio decreased to US$19.19. The wider vocational rehabilitation population had cost-wage ratios of US$12.01 and US$9.73 during the same period. In relation to costs per hours worked, comparable cost trends were found. When the cost per wages earned was compared, ASD was more costly to serve than the other conditions included in the study, such as traumatic brain injury, mental illness and learning disabilities. It was also reported that the employment rate for individuals with ASD was 40.85%. The only other areas of disabilities that experienced higher rates of employment were people with learning disabilities (41.8%) and sensory impairments (57.2%). This shows that while ASD was one of the most expensive groups to put through vocational rehabilitation services, it was more efficient for adults with ASD, rather than being unemployed, compounding an already significant cost. This study also indicates that individuals with ASD have a strong chance of becoming employed once they have appropriate supports, thereby having worthwhile investment potential for vocational rehabilitation services.

Jӓrbrink and Knapp (45) explored the implications of the cost of ASD to the UK. After assuming 5 per 10,000 people experience ASD, they estimated annual UK societal costs were more than £1 billion. The individual with ASDs’ lifetime cost was greater than £2.4 million. The major costs were living support and day activities. Costs for families represented 2.3% of the total cost of ASD to the UK. The lifetime cost of placing an adult with ASD in sheltered work was £16,200, 0.6% of the total cost ASD. The total lifetime cost of placing an adult with ASD in sheltered employment was 8.6% of the total cost. This highlights the significant lifetime cost (£67,800) for society to place an individual who has ASD in a sheltered workplace, when alternatively employing these individuals who have specific skills and abilities, would save UK taxpayers £67,800 per individual with ASD over a lifetime.

Ganz (37) explored consequences for society for overlooking employing adults with ASD. Using a hypothetical ASD cohort, the study aimed to define both costs over a lifetime of ASD and age specific costs in US. The findings from this study were that lifetime societal cost of ASD amounted to US$3.2 million per capita. It was also found that lost productivity and adult care were the largest contributors of these costs. Therefore, employing adults with ASD would significantly reduce the lifetime cost of ASD in terms of lost productivity. In addition, employing individuals with ASD would decrease the reliance on adult care or daily activities, ultimately significantly reducing these costs to society.

#### Employer benefits

A total of three studies explored the costs of employing an adult with ASD. Schaller and Yang (46), examined whether people with ASD receiving competitive employment services were statistically significantly diverse compared to individuals with ASD receiving supported employment services. This was completed in relation to successful closure rates for their vocational rehabilitation cases, hours worked per week, earnings per week and average case service cost. The average hours worked competitively per week by participants was 27.19 (SD = 11.36), and the average hours worked for the supported employment participants was 22.21 (SD = 10.33), which showed a significant difference between the groups (t = 5.31; p < .001). They also found that the mean cost of services for competitive employment participants was US$3,341 (*SD* = US$5,744.); while the supported employment participants was US$6,883 (SD = US$9,497), which was a significant difference (t = 6.65; p < .001) [46]. This study identified information on important factors that are involved in a cost-benefit ratio in terms of weekly average hours worked by the group in competitive employment (27.19). The results demonstrate that individuals with ASD can continuously contribute at a worksite for a significant period of time.

Cimera and Burgess (42) aimed to understand if working in the community was cost-efficient from the perspective of an employee with ASD. They found that not only working in the community was cost effective from the perspective of the employee with ASD, but also that their hours worked per week were consistent during 2002–2007 (mean hours = 23.7/week). This study showed that adults with ASD not only receive benefits from working competitively, but can provide benefits to the employer, specifically by maintaining consistent hours worked per week for significant periods of time.

Burgess and Cimera (39) evaluated the employment outcomes for adults with ASD, who had used vocational rehabilitation providers during 2002–2011. The findings were that during this period the amount of hours worked per week (22–26) by individuals with ASD was consistent across the states of the US. It was reported that the number of adults using vocational rehabilitation services had increased during the past 10 years from a low of 913 individuals representing 0.86% of the total amount of people receiving vocational rehabilitation services in 2002 to 8,154 which accounts for 5.43% of the group in 2011. These findings demonstrate two points; that again, there is information that adults with ASD can contribute to a workplace for a significant number of hours per week over an extended period across a country, as well showing that an increasing number of adults with ASD are using vocational rehabilitation services seeking employment, demonstrating a desire and a willingness to work and contribute, which would interest employers who are looking for reliable employees who want to really contribute to their business.

## Discussion

Several consistent points from the current knowledge base emerged. It is clear that there was a significant decrease in the number of benefits governments had to pay to adults with ASD once they were employed [47, 48]. The total lifetime cost of placing an adult with ASD in sheltered employment was 8.6% of the total cost [45]. This again highlights the increase of lifetime cost to society if interventions focus on placing individual with ASD in a sheltered workplace instead of interventions aimed at open or supported employment for adults with ASD. These results strongly indicate that governments can make savings by supporting employment services for adults with ASD. These services do not only reduce the cost for governments compared with providing standard care, they will also result in better outcomes for adults with ASD.

Unemployment and underemployment of adults with ASD may also be considered as an expensive overlooked opportunity, since it results in lost productivity and a demand for services providing adult care [37]. Hence, providing employment opportunities for adults with ASD enables this group to contribute valuable services to the society, while reducing costs for daily activities [32]. Considering the estimated annual societal cost for adult care and daily activities in the UK was more £1 billion, the results from the current review showed that by initially spending money on supporting individuals with ASD to get into employment, governments can save significant costs through increased productivity, reduced amount of benefits, and less required funding for daily activities and community supports. Furthermore, despite the fact that ASD was the most expensive group to provide vocational rehabilitation services for, it appears that adults with ASD have a strong chance of becoming employed once appropriate measures are in place. As a consequence, rehabilitation services could also be considered as a worthwhile investment [40].

The current systematic review only found a few studies that explored the economic benefits of employing individuals with ASD for employers specifically. Three studies found similar results regarding the number of hours per week adults with ASD were able to work consistently over a period of years. These studies showed that adults with ASD can, on average, contribute 23.30 hours per week. Although this information is relevant to employers as they may be more likely to employ individuals that they consider reliable and will be able to work for a consistent period, more studies are needed, in order to provide employers with information that may enhance their inclination to employ individuals with ASD. The fact that there is a potential to greatly reduce societal costs, as of yet it is probably not a strong enough incentive for individual employers to employ adults with ASD.

It could be concluded that enhancing the opportunities for adults with ASD to join the workforce is beneficial from a societal perspective, not only from an inclusiveness viewpoint, but also from a strict economic standpoint. However, the current systematic review uncovered the fact that very few studies have examined the benefits, the costs and the cost-benefit ratio of employing an adult with ASD from the perspective of employers. It is obvious that there is a significant need for this topic to be further explored from the perspective of employers. Increased knowledge about costs and benefits of employing adults with ASD may show employers that the benefits of employing adult with ASD outweigh the cost. Furthermore, additional research into benefits for employers, including the greater work ethic and better focus that individuals with ASD may apply to roles and jobs is warranted.

The results of this study are relevant to occupational therapy practice for adults with ASD and can be best explained through The Triangle of Health and Wellbeing developed by Wilcock (49) which recognizes that a key contributor to well-being and health for humans is occupational participation. This framework defines *doing* as the act of working towards meeting basic human needs to improve health and well-being. Having this internal motivator to engage in *doing* with others for a shared purpose provides a sense of belonging and purpose [49]. It is this *doing* and *belonging* that leads to improving and growing a persons’ well-being and their health. Occupational therapy focuses on providing ways for individuals with ASD to engage in their meaningful activities, such as employment (*doing*). However, by providing the opportunities for these adults to become employed, occupational therapists need to be confident that employers are equipped to manage this working relationship, so that it can be mutually beneficial. Further research that explores not only the employers’ perspective but also the perspective of employees with ASD could inform therapists to support employers in create a work environment that enables adults with ASD to work at their full capacity. Furthermore, it may create awareness that employment of adults with ASD is important and potentially essential to certain companies.

The fact that so few studies were available is a limitation of the current systematic review. In total, only three countries are represented, i.e., U.K, USA and Sweden. Although theses counties are relatively equal in relation to GDP per capita (2014 the World Bank reported that in US $ GDP/ capita was; 45,603 in U.K, 54.630 in USA and 58,887 in Sweden), the labour markets and service systems differ. Furthermore, the studies were conducted between the years 2005–2014. Consequently, some findings may be outdated due to changes related to employment and service provision in the three countries. Hence, a true cross national comparison of cost and benefits was not possible due to lack of data. The results of the current review should therefore be interpreted with due caution and may only apply to countries with similar economic structures. However, it can be concluded that in each of these countries employing adults with ASD in competitive employment was economically beneficial on a societal level. Furthermore, it may be concluded that by creating competitive employment opportunities for individuals with ASD, the social capital of a society is probably strengthened. Social capital is the network of relationships among people living and working within a particular society, enabling that society to function effectively and in cohesion [50].

## Conclusion

It could be concluded that enhancing the opportunities for adults with ASD to join the workforce is beneficial from a societal perspective, not only from an inclusiveness viewpoint, but also from a strict economic standpoint. Governments can ultimately make savings by spending money on providing supported employment services for adults with ASD. These services do not only cut the cost for governments compared with providing standard care, they will also results in better outcomes for adults with ASD. Furthermore, despite the fact that adults with ASD are the most expensive group to provide vocational rehabilitation services for, it appears that they have a strong chance of becoming employed once appropriate measures are in place, indicating that rehabilitation services could be considered as a worthwhile investment [40]. However, the current systematic review uncovered the fact that very few studies have examined the benefits, the costs and the cost-benefit ratio of employing an adult with ASD from the perspective of employers. Furthermore, existing service system and governmental subsidiaries could be expected to significantly impact an employers’ inclination to employ adults with ASD. However, these systems vary between the nations represented in the included articles. Based on the current review it is therefore not possible to make a conclusion about costs and benefits of employing an adult with ASD from the perspective of employers. It is obvious that there is a significant need for this topic to be further explored from this perspective.

## Supporting Information

S1 Table The Cost Effectiveness of Employing Adults with ASD to Governments, Society and Employers. The Level number under design refers to the level of evidence for articles where; level I = A systematic review of level II studies or large multicentre trial, level II = A randomised controlled trial, level III = A quasi-experimental study, cohort or case control, level IV = A pre-experimental, pre-test post-test, correlational studies of multiple sites, level V = Single site correlational studies, descriptive studies, qualitative studies, expert opinion [12].
(DOCX)

S2 Table The Kmet Checklist. This list was used to access the scientific quality of the included articles. (DOCX)

S3 Table The data extraction form. The form is based on The Cochrane Handbook for Systematic Reviews Section 7.3. It comprise the headings and the outcomes variables used as a guide for data extraction.
(DOCX)
