# Supplementary material for: The Costs and Benefits of Employing an Adult with Autism Spectrum Disorder: A Systematic Review
Source: PLoS One. 2015 Oct 7;10(10):e0139896. doi: 10.1371/journal.pone.0139896 (PMC4596848; doi:10.1371/journal.pone.0139896)
Supplement: S1 Table — The Level number under design refers to the level of evidence for articles where; level I = A systematic review of level II studies or large multicentre trial, level II = A randomised controlled trial, level III = A quasi-experimental study, cohort or case control, level IV = A pre-experimental, pre-test post-test, correlational studies of multiple sites, level V = Single site correlational studies, descriptive studies, qualitative studies, expert opinion [12]. (DOCX) [file pone.0139896.s001.docx]

**S1 Table. The Cost Effectiveness of Employing Adults with ASD to Governments, Society and Employers.** The Level number under design refers to the level of evidence for articles where; level I= A systematic review of level II studies or large multicentre trial, level II= A randomised controlled trial, level III= A quasi-experimental study, cohort or case control, level IV= A pre-experimental, pre-test post-test, correlational studies of multiple sites, level V= Single site correlational studies, descriptive studies, qualitative studies, expert opinion [12].

| **Population** | **Design** | **Aims** | **Results** | **Kmet score** | **Kmet summary** |
| --- | --- | --- | --- | --- | --- |
| 1 [[41](#_ENREF_41)] UK.  203 participants were diagnosed with ASD or Asperger syndrome and had been involved in a supported employment program during an 8 year period. | Cohort study (retrospective)  (Level 3) | Explores over an 8 year period the findings about a supported employment services for adults with ASD. | Found that the cost per job reduced from £6542 in 2000–1 to £4281 in 2002–3.  Total saving to the Exchequer in reduction of benefits paid and gains in tax and national insurance, was £ 179, 095 for the 114 jobs found from programme.  A significant decrease was found in the number of benefits received once adults became employed (median pre-work = £2907, range £0–£9193; median post-work = £0, range £0–£6801; median reduction = –£1974, range +£1440 to –£9030; Wilcoxon *Z* = –7.72; *p* < 0.001). | 82%  (Strong) | Well described participants and results.  Adequate sample size.  No mention of controlling for confounding factors. |
| 2 [[46](#_ENREF_46)] USA.  A group of 450 participants were people with ASD who entered competitive employment services and a group of 365 participants with ASD who entered supported employment services, totalling 815 participants. | Correlational  (Level 5) | To explore if people with ASD receiving competitive employment services are  significantly diverse from individuals  with ASD who entered supported employment services in terms of successful closure rates for their vocational rehabilitation cases, earnings per week, hours worked per week and average case service cost.  Understanding if the participants case service variables were statistically significantly correlated to successful closure in competitive employment for individuals with ASD who obtained/did not obtain supported employment services. | The total positive competitive employment closure rate for individuals was 263 out of 450 (58.4%). Individuals in supported employment obtained 275 from 365 (75.3%), (2 = 25.64; *p* = .00)  Average hours worked competitively per week by participants was 27.19 (SD = 11.36). Average hours worked by the supported participants was 22.21 (SD = 10.33), (t = 5.31; p = .00).  Average weekly earnings at point of closure for the competitive employment group was US$205.31 (SD = US$132.90). The group in supported employment having average weekly earnings of US$138.35 (SD = US$84.87), (t = 6.99; p = .00).  Mean cost of service for competitively employed participants employed was US$3,341.14 (*SD* =US$5,744.). Mean cost of service for the supported employment participants was US$6,882.46 (SD = US$9,497), (t = 6.65; p = .00).  Within the competitive employment group a person’s increased age, lack of secondary disability, successful job placement and obtaining maintenance services were statistically significant factors in successful completion. | 95%  (Strong) | Appropriate sample size and description of participants.  Analytic methods appropriate and results well described. |
|  | S1 Table. (continuing) | | | | |
| **Population** | **Design** | **Aims** | **Results** | **Kmet score** | **Kmet summary** |
| 3 [[32](#_ENREF_32)] Sweden.  Between 2000 and 2003 a study was done in four communities  in western Sweden.  Between 2001/  2002 and 2002/2003, information was gathered  from relatives of 19 adults with ASD. | Descriptive  (Level 5) | The aim was to enhance the evidence base for individuals with ASD by improving understanding about ASD for authorities who can assist individuals with ASD search for employment. | The average annual community support cost for each adult with ASD was €7154.  Employment support and project support accounted for 4.0% of the total annual cost. Community support represented 22.6% of total annual cost (€596 per month per participant). The cost of daily activities accounted for 20.9% of the baseline total service cost or €310 per person per month.  The employment services cost represented 2.6% of the total cost for these participants.  Average annual informal care cost was estimated at €1554 with expenses at €1052. Informal care costs represented 8.2% of total cost. | 68%  (Adequate) | Study used qualitative interviews with relatives to obtain information around costs which may not have been appropriate analytic methods. Study was limited to four communities in Sweden.  Participants were only broadly described.  No mention of confounding factors was mentioned in study. |

|  | S1 Table. (continuing) | | | | |
| --- | --- | --- | --- | --- | --- |
| **Population** | **Design** | **Aims** | **Results** | **Kmet score** | **Kmet summary** |
| 4 [[42](#_ENREF_42)] USA.  During 2002 - 2007, 19,436 of these individuals who experience ASD who applied for services via state vocational rehabilitation agencies. | Case –Control (retrospective)  (Level 3) | To explore if working in the community was  cost-efficient from the employee’s viewpoint?  To explore if employment outcomes during 2002 to 2007, improved.  Aimed to understand if individuals who did not experience  secondary conditions achieved different  employment outcomes.  To see if employment outcomes varied across the US. | Individuals with ASD working in the community generated more financial benefits than financial costs (Average benefit-cost ratio of 5.28) (Monthly net benefit of US$643.20).  Employment rates (*M*= 40. 6%), hours worked per week (*M*= 23.7), and wages earned per month (*M*= US$793.34) throughout US during 2002 - 2007. | 95%  (Strong) | Appropriate description of participants.  Sample size appropriate and analytic methods well described.  Controlled for confounding factors such as secondary conditions. |
| 5 [[43](#_ENREF_43)] USA. |  |  |  |  |  |
| Two groups were compared. A group of 215 supported employees from sheltered workshops and 215 supported employees who were not in sheltered workshops previously | Case Control  (Level 3) | To assess if people with ASD, enrolled in sheltered workshops before going into supported employment programs, would have better outcomes who did not receive sheltered employment services | No differences were found in inter-group employment rates. Adults previously in sheltered workshops earned less (US$129.36 compared to US$191.42 per week), and cost more to serve (US$6,065.08 compared to US$2,440.60), as opposed to the group who had not been in sheltered workshop prior to taking up supported employment.  Individuals with ASD had better vocational outcomes by not enrolling in a sheltered workshop before entering supported employment. | 100%  (Strong) | Appropriately described subjects and sample size.  Reported variance within study.  Controlled for confounding. |

|  | S1Table. (continuing) | | | | |
| --- | --- | --- | --- | --- | --- |
| **Population** | **Design** | **Aims** | **Results** | **Kmet score** | **Kmet summary** |
| 6 [[38](#_ENREF_38)] UK.  A modular approach was used due to the nonexistence of a national data source on ASD in the UK, for estimating: characteristics, age, prevalence, place of residence, cost per person. | Descriptive  (Level 5) | To estimate the economic consequences of ASD in the UK. | Mean annual costs (including lost employment, but excluding benefits) were for an intellectually disabled adult with ASD residing in family households (£36,507), in supported accommodation (£87,662), in residential care (£88,937) and for long term hospital care (£97,863).  It was found that for a non-intellectually disabled adult with ASD, living in a family household, annual cost was £ 32,681, with a major element being lost productivity for society and tax revenue for the Exchequer and the person’s lost employment.  It was found the aggregate national UK cost for adults with ASD was £25 billion (excluding benefit payments). The cost of supporting intellectually disabled adults (including lost employment) represents two-thirds of these costs (£17 billion). Publicly funded services accounted for 59% of this total, with lost employment for the person (36%) and family expenses (5%) representing the rest.  The discounted lifetime cost for individuals with ASD who did not experience intellectual disability was £796,050, (undiscounted lifetime cost was £3.1 million).  The lifetime cost for a person with ASD who also experienced an intellectual disability was found to be 50% higher at £1,234,044 (discounted) (£ 4.6 million undiscounted).  The total estimated UK economic cost of ASD was £28 billion, which amounted to £500 for every adult and child in the UK annually. | 83% (Strong) | Well described outcomes and analytic methods and results reported in sufficient detail.  No mention of estimates for variance. |

|  | S1 Table. (continuing) | | | | |
| --- | --- | --- | --- | --- | --- |
| **Population** | **Design** | **Aims** | **Results** | **Kmet score** | **Kmet summary** |
| 7 [[39](#_ENREF_39)] USA.  Participants included 34, 501 transition-aged adults with ASD in the US who had their VR cases finalised during 2002-2011 | Cohort study (retrospective)  (Level 5) | To evaluate the employment outcomes of transition aged adults who experience ASD served by vocational rehabilitation providers during the last ten years. | The amount of transition – aged adults with ASD who enrolled in vocational rehabilitation services in the US increased during the past 10 years from a low of 913 individuals representing 0.86% of the total amount of people receiving vocational rehabilitation services in 2002 to 8,154 which accounts for 5.43% of the group in 2011.  A mean total of 36% of transition-aged adults with ASD were employed via vocational rehabilitation services across the states of the US during the last 10 years.  During this time period the number of hours worked per week by this group was stable (22-26) across the states of the US.  The mean cost of providing vocational rehabilitation services to these adults with ASD during 2002-2011 was US$2,437 | 95%  (Strong) | Used appropriate methods.  Participants described well.  Results reported well.  No mention of controlling for confounding factors |
| 8 [[44](#_ENREF_44)] UK.  The total number of participants was (*n* = 50) The intervention group was made up of individuals with a formal diagnosis of ASD all were seeking work. Control group was made up adults with ASD with similar linguistic and intellectual ability. | Case control  (Level 3) | To explore the cost-benefit of day services versus supported employment for adults with ASD in the UK. | Supported employment for adults with ASD was found to have better outcomes in comparison to standard care, at increased cost of £5600 per quality-adjusted life year or £18 per additional week in employment. | 64%  (Adequate) | Appropriately described participants.  No mention of random allocation for participants.  Sample size was limited (n = 50).  No mention of controlling for confounding factors. |

|  | S1 Table. (continuing) | | | | |
| --- | --- | --- | --- | --- | --- |
| **Population** | **Design** | **Aims** | **Results** | **Kmet score** | **Kmet summary** |
| 9 [[40](#_ENREF_40)] USA.  11,569 participants who were diagnosed with ASD and had their vocational rehabilitation cases closed during the 2002 – 2006. | Cohort Study  (retrospective)  (Level 3) | To explore the cost of services and employment outcomes for adults with ASD within the US vocational rehabilitation system. | There was an increase in the number of adults with ASD enrolling in vocational rehabilitation services during 2002-2006, from 1534 people to 3397 people.  During 2002, the cost of providing vocational rehabilitation services to an individual with ASD was US$3282 per person. In 2006 this had decreased to US$2992. This was in comparison to the costs of providing the same services to the overall vocational rehabilitation population, which conversely increased from US$2263 to US$2336 in this time.  The wages earned by adults with ASD, during 2002 this group cost the vocational rehabilitation US$26.74 for every dollar these individuals earned in wages. In 2006 this ratio decreased to US$19.19. The wider vocational rehabilitation population had cost-wage rations of US$12.01 and US$9.73 during the same period. Comparable cost trends for costs per hours worked was also found.  In comparison to other conditions, people with ASD were amongst the most costly of nine conditions studied.  Comparing costs within the ASD population, individuals who only experienced ASD cost vocational rehabilitation services US$3002. This was opposed to individuals who experienced ASD with other conditions who cost US$6141.  The employment rate for these individuals who experienced ASD was 40.85%. Only people with learning disabilities (41.8%) and sensory impairments (57.2%) had higher rates of employment. | 95%  (Strong) | Study design was appropriate and subjects well described.  Sample size was appropriate.  No mention of controlling for confounding factors. |

|  | S1 Table. (continuing) | | | | |
| --- | --- | --- | --- | --- | --- |
| **Population** | **Design** | **Aims** | **Results** | **Kmet score** | **Kmet summary** |
| 10 [[45](#_ENREF_45)]UK.  228 participants who had been d diagnosed with ASD. | Descriptive  (Level 5) | Explore the cost implications of ASD | Assuming 5 per 10,000 people have ASD, the estimated annual UK societal cost was more than £1 billion.  An individual with ASD’s lifetime cost was greater than £2.4 million. Major costs were living support and day activities. Costs to the family represented 2.3 % of the total cost of ASD to the UK.  The lifetime cost of placing an adult with ASD in sheltered work was £16,200, 0.6% of the total cost ASD. The total lifetime cost of placing an adult with high functioning ASD in sheltered employment was £67,800 or 8.6% of the total cost. | 80%  (Strong) | Subjects described in detail.  Sample size appropriate.  Analytic methods partially described.  No mention of variance or SD.  Results reported in sufficient detail. |
| 11 [[37](#_ENREF_37)] USA.  A hypothetical ASD cohort born in 2000 and diagnosed in 2003. | Cohort study  (Level 3) | To describe age-specific and lifetime costs of ASD in the US. | The lifetime societal cost of ASD was US$3.2 million per capita. Adult care and lost productivity were the largest components of costs. | 64%  (Adequate) | Methodology partially appropriate.  Subjects well described.  Sample size hypothetical and may not be appropriate.  Analytic methods well described. |
