# Supplementary material for: The Costs and Benefits of Employing an Adult with Autism Spectrum Disorder: A Systematic Review
Source: PLoS One. 2015 Oct 7;10(10):e0139896. doi: 10.1371/journal.pone.0139896 (PMC4596848; doi:10.1371/journal.pone.0139896)
Supplement: S2 Table — (DOCX) [file pone.0139896.s002.docx]

**S2 Table. The Kmet Checklist.** This list was used to assess the scientific quality of the included articles.

| **Number** | **Criteria** | **Yes** | **Partial** | **No** | **N/A** |
| --- | --- | --- | --- | --- | --- |
| **1** | **Question/objective sufficiently described?** |  |  |  |  |
| **2** | **Study design evident and appropriate?** |  |  |  |  |
| **3** | **Method of subject/comparison group selection or source of information/input variable described and appropriate?** |  |  |  |  |
| **4** | **Subject and (comparison group, if applicable) characteristics sufficiently described?** |  |  |  |  |
| **5** | **If interventional and random allocation was possible, was it reported?** |  |  |  |  |
| **6** | **If interventional and blinding of investigators was possible, was it reported?** |  |  |  |  |
| **7** | **If interventional and blinding of subjects was possible, was it reported?** |  |  |  |  |
| **8** | **Outcome and (if applicable) exposure measure(s) well defined and robust to measurement/misclassification bias? Means of assessment reported?** |  |  |  |  |
| **9** | **Sample size appropriate?** |  |  |  |  |
| **10** | **Analytic methods described/justified and appropriate?** |  |  |  |  |
| **11** | **Some estimate of variance is reported for the main results?** |  |  |  |  |
| **12** | **Controlled for confounding?** |  |  |  |  |
| **13** | **Results reported in sufficient detail?** |  |  |  |  |
| **14** | **Conclusions supported by the results?** |  |  |  |  |
