# Supplementary material for: The Costs and Benefits of Employing an Adult with Autism Spectrum Disorder: A Systematic Review
Source: PLoS One. 2015 Oct 7;10(10):e0139896. doi: 10.1371/journal.pone.0139896 (PMC4596848; doi:10.1371/journal.pone.0139896)
Supplement: S3 Table — It comprise the headings and the outcomes variables used as a guide for data extraction. (DOCX) [file pone.0139896.s003.docx]

**S3 Table. The data extraction form.** The form is based on The Cochrane Handbook for Systematic Reviews Section 7.3. It comprise the headings and the outcomes variables used as a guide for data extraction

| **Headings** | Data extracted from: |
| --- | --- |
| Citation |  |
| Publication status |  |
| Database |  |
| Level of evidence |  |
| Study design |  |
| Population |  |
| Methods |  |
| **Outcomes were grouped depending on if the study displayed** | |
| The cost effectiveness of employing an adult with autism spectrum disorder to governments |  |
| The cost effectiveness of employing adults with autism spectrum disorder to society |  |
| The cost effectiveness of employing of employing these adults with autism to employers |  |
| Results |  |
